# 

**Published:** 1882-11

**Authors:** 


### CONTENTS:

| | PAGE. |
|---|---|
| **Original Communications:** | |
| Histology of the Nerves: a Contribution of the Latest Researches. By Frank Dudley Beane, A. M., M. D., | 145 |
| Secondary or Recurrent Hemorrhage. By Charles C. F. Gay, M. D., | 159 |
| **Selections:** | |
| Narcotism in Infancy, | 167 |
| Fowler's Solution. By E. C. Seguin, | 169 |
| Inebriety and the Use of Opium, | 173 |
| Traumatic Tetanus and Death from Vaccination, | 174 |
| Floating Kidneys, | 174 |
| Varicose Veins, | 175 |
| Koch on Disinfectants, | 175 |
| The Odor of Iodoform, | 176 |
| Hypophosphites in Phthisis Pulmonalis, | 176 |
| A Fistula Knife. | 177 |
| Titles of Specialists, | 177 |
| Amussat's Laxative Syrup, | 177 |
| **Society Reports:** | |
| Buffalo Medical and Surgical Association, | 178 |
| **Editorial:** | |
| The Prevailing Sentiment on the Consultation Question, | 186 |
| Editorial Notes, | 187 |
| **Reviews:** | 188 |

